# *Vigna yadavii* (Leguminosae: Papilionoideae), a new species from Western Ghats, India

**DOI:** 10.3897/BDJ.2.e4281

**Published:** 2014-12-23

**Authors:** Sayajirao P. Gaikwad, Ramchandra D. Gore, Sonali D. Randive, Krushnadeoray U. Garad

**Affiliations:** † Life Science Research Laboratory, Walchand College of Arts and Science, Solapur- 413 006 (MS), Solapur, India

**Keywords:** *Ceratotropis*, dimorphic seeds, taxonomy.

## Abstract

A new species of *Vigna* Savi, subgenus *Ceratotropis* (Piper) Verdc., *Vigna yadavii* S.P. Gaikwad, R.D. Gore, S.D. Randive & K.U. Garad, **sp. nov.** is described and illustrated here. It is morphologically close to *Vigna dalzelliana* (Kuntze) Verdc. but differs in its underground obligate cleistogamous flowers on positively geotropic branches, hairy calyx, small corolla, linear style beak and dimorphic seeds with shiny seed coat.

## Introduction

*Vigna* Savi is a large pantropical genus of the tribe Phaseoleae with 90 species distributed in six subgenera (Thulin et al. 2004, Delgado-Salinas et al. 2011, Tomooka et al. 2010). Among the subgenera of the genus *Vigna* only the subgenus *Ceratotropis* (Piper) Verdc. has its center of species diversity in Asia and it is popularly known as Asian *Vigna* (Tomooka et al. 2002b). The subgenus *Ceratotropis* comprises about 22 species in all three sections viz. *Aconitifoliae* Tomooka & Maxted, *Angulares* Tomooka & Maxted and *Ceratotropis* (Piper) Verdc. (Tomooka et al. 2002a, Tomooka et al. 2010). However, Aitawade et al. (2012) have described a new species *V. sahyadriana* Aitawade, K.V. Bhat & S.R. Yadav from India recently. Thus, the number of species in the genus *Vigna* subgenus *Ceratotropis* is now 23.

During field survey of plants of the family Leguminosae – Papilionoideae in Western Ghats of India, the authors collected an interesting species of *Vigna* on hill slopes at about 1200 m elevation above mean sea level in Nasik and Satara districts of Maharashtra, India. It interestingly possesses underground cleistogamous flowers on positively geotropic branches. This unusual character of *Vigna* species encouraged its detailed study, which revealed that it represents an un-described species of the genus *Vigna* subgenus *Ceratotropis*. It has been confirmed by the perusal of relevant literature (Maréchal et al. 1978, Tateishi 1984, Babu et al. 1987, Tomooka et al. 2002a, Tomooka et al. 2002b, Tomooka et al. 2010, Maxted et al. 2004, Thulin et al. 2004, Lewis et al. 2005, Delgado-Salinas et al. 2011, Aitawade et al. 2012 and Aitawade et al. 2012) and experts' opinion on the identity of the species. It is described and illustrated here.

## Taxon treatments

### Vigna yadavii

S.P. Gaikwad, R.D. Gore, S.D. Randive & K.U. Garad. 2014 sp. nov.

urn:lsid:ipni.org:names:77144279-1

#### Materials

- **Type status:** Holotype. **Location:** continent: Asia; country: India; countryCode: IND; stateProvince: Maharashtra; municipality: Nasik district; locality: Kasara-Ghat near Igatpuri; verbatimElevation: 365 m; verbatimLatitude: 19°41'02.1"N; verbatimLongitude: 73°29'58.3"E; verbatimCoordinateSystem: degrees minutes seconds; **Identification:** identifiedBy: N. Tomooka; M. Sanjappa; Delin Wu; **Event:** eventDate: 10-11-2012; habitat: Western Ghats; fieldNumber: RD Gore 1042; fieldNotes: Twining herbs; leaves stipulate; stipules submedifixed; chasmogamous flowers yellow & Cleistogamous flowers white. Pods falcate to straight. Seeds well developed; **Record Level:** type: Herbarium Specimen; language: English; institutionID: CAL
- **Type status:** Isotype. **Location:** continent: Asia; country: India; countryCode: IND; stateProvince: Maharashtra; municipality: Nasik district; locality: Kasara-Ghat near Igatpuri; verbatimElevation: 365 m; verbatimLatitude: 19°41'02.1"N; verbatimLongitude: 73°29'58.3"E; **Event:** eventDate: 10-11-2012; habitat: Western Ghats; fieldNumber: RD Gore 1042a; fieldNotes: Twining herbs; leaves stipulate; stipules submedifixed; chasmogamous flowers yellow & Cleistogamous flowers white/albino. Pods falcate to straight. Seeds well developed; **Record Level:** language: English; institutionID: BSI, Pune
- **Type status:** Other material. **Location:** continent: Asia; country: India; countryCode: IND; stateProvince: Maharashtra; municipality: Nasik District; locality: Saptashrungi hills (Kalvan); **Identification:** identifiedBy: S.P. Gaikwad; R.D. Gore; **Event:** eventDate: 9-11-2012; habitat: Western Ghats; fieldNumber: RD Gore 1040; fieldNotes: Twining herbs; flowers both chasmogamous (yellow) and cleistogamous (white/albino); **Record Level:** language: English; institutionID: Walchand College of Arts & Science, Solapur
- **Type status:** Other material. **Location:** continent: Asia; country: India; countryCode: IND; stateProvince: Maharashtra; municipality: Nasik District; locality: Kasara-Ghat near Igatpuri; **Identification:** identifiedBy: S.P. Gaikwad; R.D. Gore; **Event:** eventDate: 10-11-2012; habitat: Western Ghats; fieldNumber: SD Randive 322; fieldNotes: Twining herbs; flowers yellow; pods slightly hairy; **Record Level:** language: English; institutionID: Walchand College of Arts & Science, Solapur
- **Type status:** Other material. **Location:** continent: Asia; country: India; countryCode: IND; stateProvince: Karnataka; municipality: Chickmanglur District; locality: Bhabathi–Gangamula; **Event:** eventDate: 8-10-1979; fieldNumber: KFP 9702; **Record Level:** language: English; institutionID: St. Joseph College, Bangalore
- **Type status:** Other material. **Location:** continent: Asia; country: India; stateProvince: Maharashtra; municipality: Satara District; locality: Pasarnighat; **Event:** eventDate: 21-10-2011; fieldNumber: SP Sutar 156; **Record Level:** language: English; institutionID: SUK
- **Type status:** Other material. **Location:** continent: Asia; country: India; countryCode: IND; stateProvince: Maharashtra; municipality: Pune District; locality: Parvati; **Event:** eventDate: 5-8-1960; fieldNumber: KNS 64502; fieldNotes: Common; **Record Level:** language: English; institutionID: BSI, Pune
- **Type status:** Other material. **Location:** continent: Asia; country: India; countryCode: IND; stateProvince: Maharashtra; municipality: Pune district; locality: Shivneri fort; **Event:** eventDate: 10-10-1962; fieldNumber: Rolla Rao 83523; **Record Level:** language: English; institutionID: BSI, Pune
- **Type status:** Other material. **Location:** continent: Asia; country: India; countryCode: IND; stateProvince: Maharashtra; municipality: Sangli District; locality: Dandoba hills (Miraj); **Event:** eventDate: 28-9-1989; fieldNumber: AN Londhe 170037; **Record Level:** language: English; institutionID: BSI, Pune

#### Description

**Figure 1.**
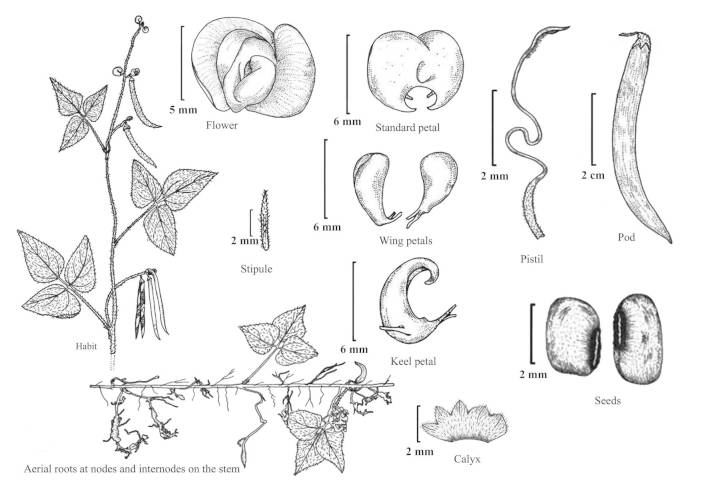
*Vigna yadavii*: chasmogamous flower [Line drawing by RD Gore; voucher *RD Gore* 1042 (CAL)].

**Figure 2.**
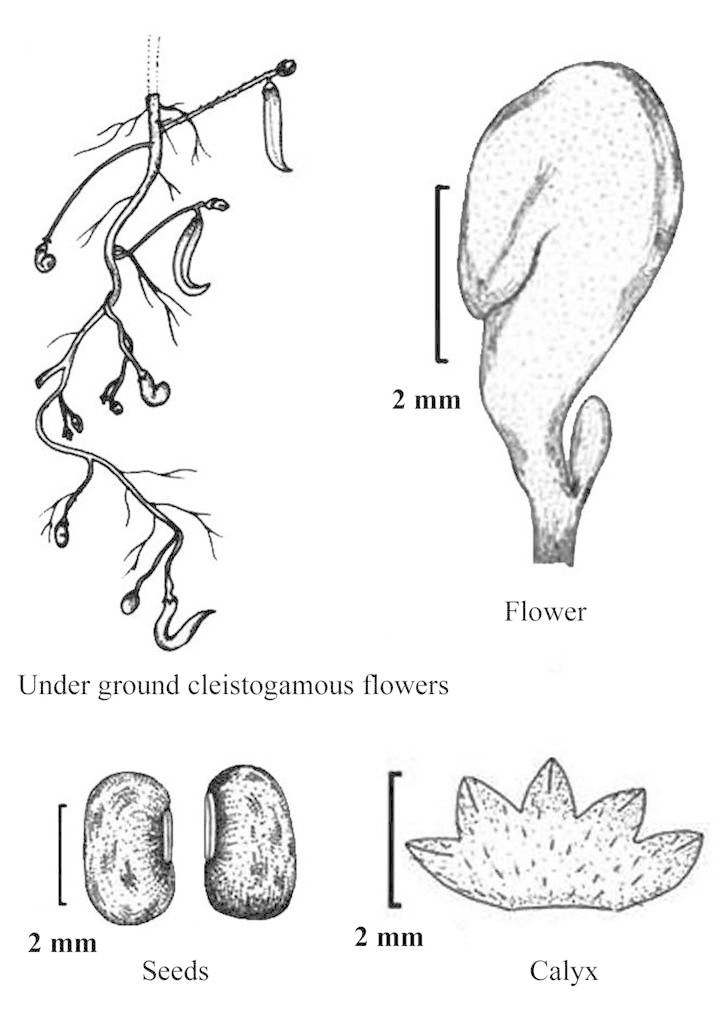
*Vigna yadavii*: cleistogamous flower [Line drawing by RD Gore; voucher *RD Gore* 1042 (CAL)].

Twining annual herbs. Stems slender, terete, 1–2 m long, covered with 1–3 mm long bulbous based spreading or retrose brownish hairs, rooting at nodes and internodes of the stem in absence of support. Stipules sub-medifixed, elliptic-lanceolate, 5–7 mm long, base obtuse to rounded, 5–7-nerved, apex acute, densely pubescent. Leaves 3-foliolate; petioles 5–7 cm long, densely hairy with bulbous based retrose or spreading whitish hairs as stem. Leaflets membranous, entire; lateral leaflets ovate-rhomboid with obliquely rounded, obtuse or truncate at base, 3–4.5 x 2–3 cm, margins entire or sometimes shallowly lobed, acute or shortly acuminate at apex, sparsely hairy; rachis 2–3 mm long, covered with whitish brown, 0.5–1 mm long, spreading or retrose hairs; terminal leaflet slightly larger than the lateral ones, ovate, 3.5–6.5 x 1.7–3.5 cm, rounded at base, shortly acuminate or acute at apex, rachis 3–4 mm long, covered with whitish-brown, 0.5–1 mm long, spreading or retrose hairs; stipels two, linear, 1–2 mm long, sparsely hairy. Flowers of two kinds, aerial chasmogamic flowers and underground cleistogamic flowers. *Chasmogamous flowers* 2–6 in axillary or terminal, lax racemes, yellow, 4.5–6 x 7–9 mm; peduncle slender, 1.5–3 cm long, densely covered with retrose whitish-brown hairs as young branches; pedicels short, 2–2.5 mm long, densely covered with whitish-brown hairs; bracts linear-lanceolate, 3–4 mm long, herbaceous, densely covered with 1–2 mm long hairs; bracteoles inserted just above the bract, linear, 3–3.5 mm long, acute at apex, densely hairy as bract. Calyx campanulate, hairy; tube c. 3 mm long; teeth triangular, 1.2 x 1 mm, sparsely hairy along margins. Standard yellow, asymmetrical, broadly ovate, 4.5–6 x 7–9 mm, emarginate at apex, central protuberance (up to 1 mm long) inside; claw c. 3 mm long. Wing petals yellow at upper portion and whitish below, 5–6 x 2.5–3 mm, membranous; right wing half concealing the upper portion of keel petals; left wing spreading horizontally and supported by a pocket on left hand keel petal. Keel petals yellowish, 5–6 mm long, spirally incurved with horn-like 1.6–2 mm long pocket, obtuse at apex. Stamens 9+1, included; staminal tube 5–6 mm long; filaments of staminal tube c. 5 mm long; free filament c. 10 mm long; anthers basifixed, 0.2–0.3 mm long. Style filiform 7–9 mm long, bearded at apex, broadly ‘S’ shaped before stigma, shortly beaked beyond the stigma; the beak linear, 0.4–0.5 mm long; ovary linear, 4.5–5 x 1–1.4 mm, minutely hairy. Pods cylindrical, 3–6 x 0.3–0.4 cm, apex acute slightly curved, sparsely hairy. Seed 6–12, rectangular, 2.5–3 x 2–2.2 mm, dark brown, mottled with black patches; seed coat shiny; hilum protruded out and well developed, elliptic, 0.9–1 mm long, white. Germination hypogeal; the first and second leaves simple, petiolate, ovate, base rounded, apex acute, sparsely hairy. *Cleistogamous flowers* 2–4 on 2–5 cm long peduncles, white (albino), 4–4.5 x 2–2.5 mm, remain closed; pedicels 1–1.2 mm long, minutely hairy; bracts elliptic–lanceolate, 1–2 mm long, acute at apex, hairy along margins with bulbous based, 0.4–0.7 mm long hairs, 1-nerved; bracteoles linear, 0.7–1.2 mm long, covered with white spreading, 0.3–0.5 mm long hairs. Calyx campanulate, membranous, c. 2.5 mm long; teeth triangular, c. 0.7 mm long, glabrescent. Standard, wing and keel petals are similar to that of chasmogamous flowers except smaller in size. Stamens 9+1, filiform; filaments 3.2–3.5 mm long; anthers basifixed, yellowish, 0.2–0.25 mm long. Style filiform, 3.2–3.9 mm long, shortly beaked beyond the stigma; beak linear, 0.2–0.3 mm long. Pods cylindrical, 1.5–2.5 cm long, usually curved, white (albino), glabrescent, apex acuminate. Seeds 3–5, whitish brown, oblong or sub-cylindric, 2.5–3 x 2–2.2 mm; seed coat shiny; hilum poorly developed, not protruded out, linear, 1–1.1 mm long, yellowish-white. Germination hypogeal; the first and second leaves simple, petiolate, ovate, elliptic, base rounded, apex acute, sparsely hairy. (Figs 1, 2, 3, 4).

##### Flowering and fruiting

August–November.

#### Diagnosis

*Vigna yadavii* is morphologically close to *Vigna dalzelliana* (Kuntze) Verdc. but differs in its underground obligate cleistogamous flowers on positively geotropic branches, hairy calyx, small corolla, linear style beak and dimorphic seeds.

#### Etymology

The species is named in honor of Prof. S.R. Yadav, Department of Botany, Shivaji University Kolhapur, India (MS), in recognition of his valuable contribution to taxonomy of flowering plants of Western Ghats of India.

#### Distribution

India, Maharashtra, Nasik Dist., Kasara-Ghat near Igatpuri and Kalvan, Saptashrungi hills; Satara Dist., Thoseghar.

#### Ecology

*Vigna yadavii* is a twining annual herb, which grows on hill slopes in grasses and herbs at about 1200 m elevation from above sea level in Western Ghats of India. The common associates of the species are *Abelmoschus manihot* (L.) Medik., *Apluda mutica* L., *Carissa congesta* Wt., *Crotalaria pallida* Ait., *C. mysorensis* Roth., *C. leptostachya* Benth., *Cymbopogon martinii* (Roxb.) Wats., *Elephantopus scaber* L., *Eragrostis* spp., *Flemingia strobilifera* (L.) R.Br. ex Ait., *Hemidesmus indicus* (L.) R.Br. ex Shult., *Themeda* spp. and *Urena lobata* L.

#### Notes

*Vigna yadavii* shows morphological similarities with *Vigna dalzelliana* (Kuntze) Verdc. but differs from the latter species by the characters given in Table 1. The presence of underground obligate cleistogamous flowers on positively geotropic branches is most useful distinguishing characteristics of *V. yadavii*. In addition to this, dimorphic seeds and differences in hilum, aril, style beak and corolla are also useful distinguishing characteristics of the new species. *Vigna dalzelliana* has a unique flattened style beak but that of *V. yadavii* is linear. The poorly developed aril of seeds of the cleistogamous flowers is also diagnostic feature of *V. yadavii*.

During rainy season (August–November), the species produces chasmogamous flowers on aerial branches and underground obligate cleistogamous flowers on positively geotropic branches. The cleistogamous flowers are much smaller than chasmogamous flowers and white-albino in color. They remain closed. The pods of cleistogamous flowers are colorless, short, curved and 3–5-seeded. There are no structural differences in chasmogamic and cleistogamic flowers except for the smaller size and white albino color of the latter.

## Supplementary Material

XML Treatment for Vigna yadavii

## Figures and Tables

**Figure 3a.**
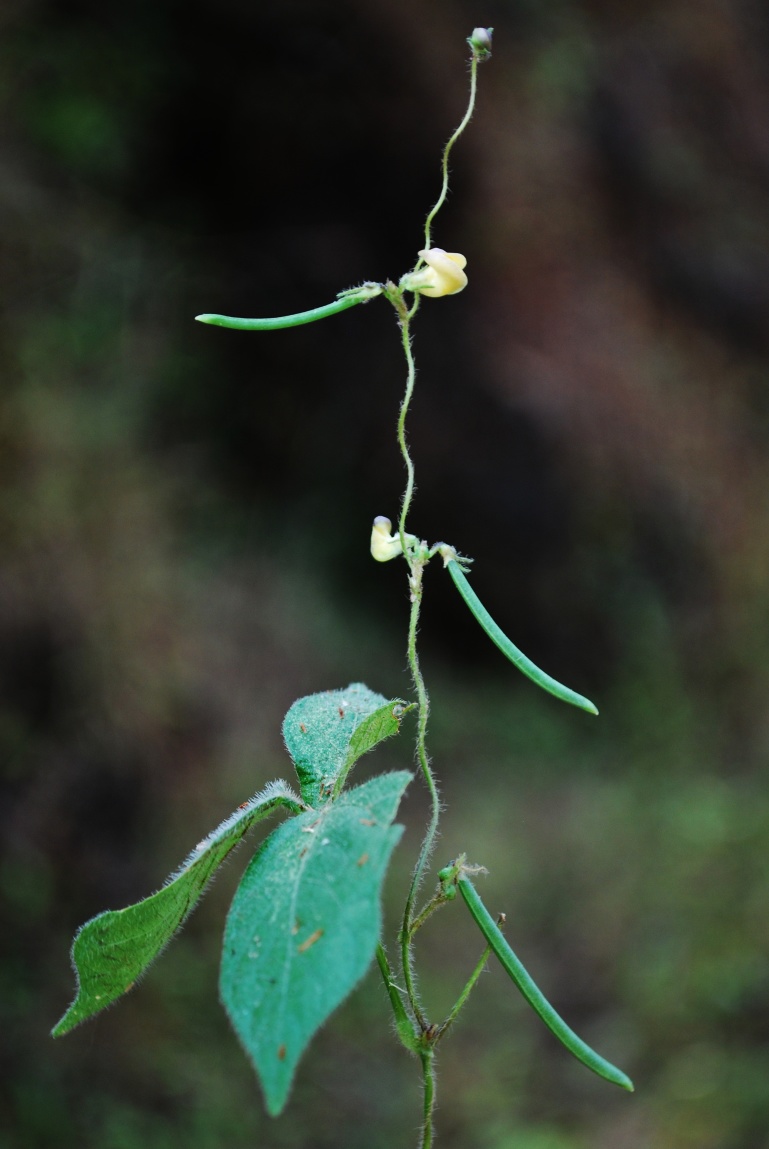
Habit

**Figure 3b.**
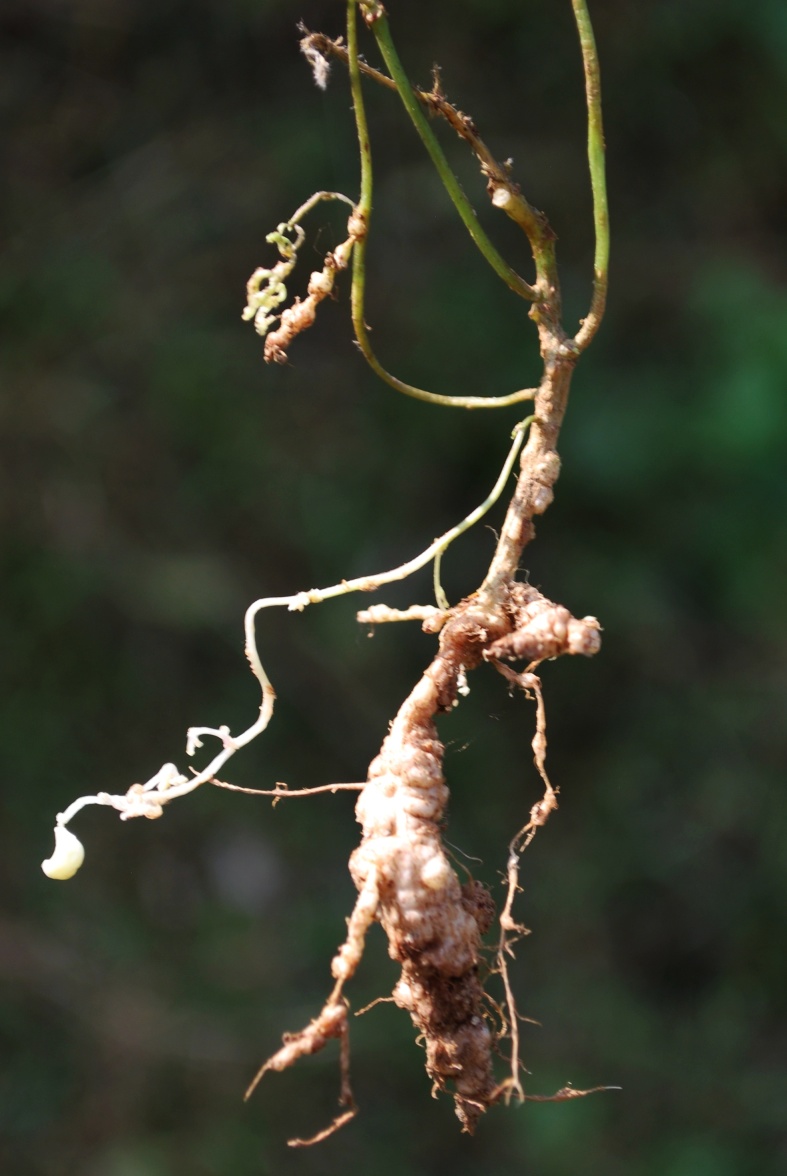
Cleistogamous flowers on positively geotropic branches

**Figure 3c.**
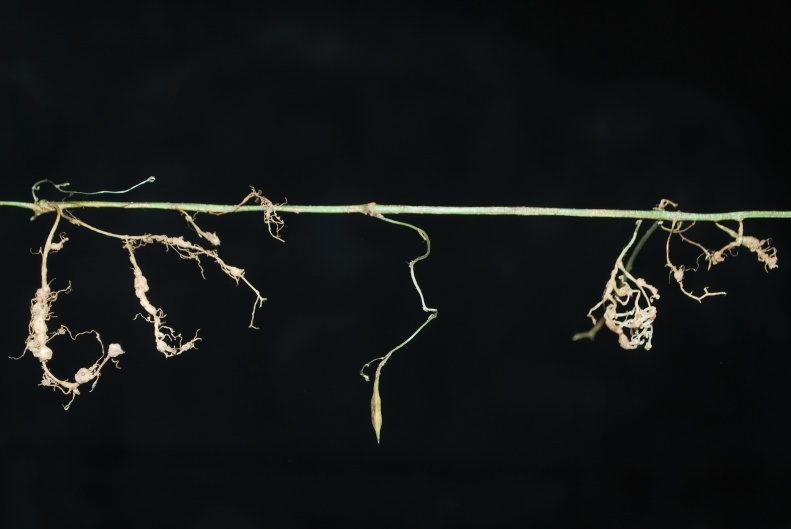
Aerial roots at nodes and internodes on the stem

**Figure 3d.**
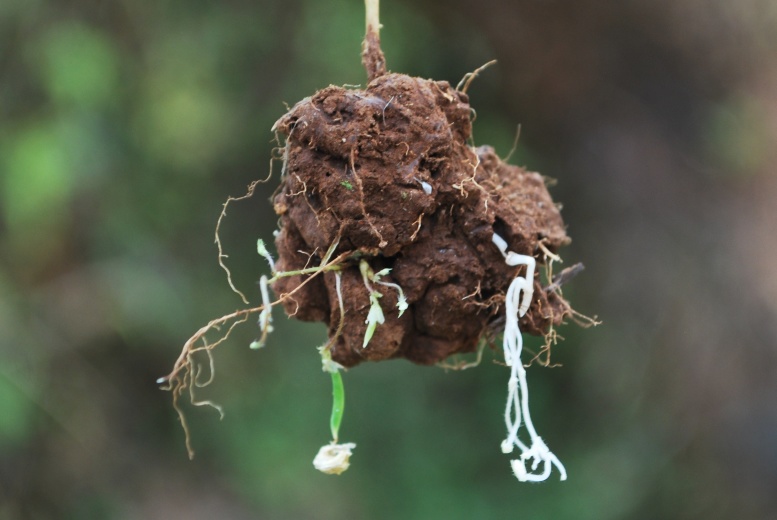
Underground cleistogamous flowers

**Figure 3e.**
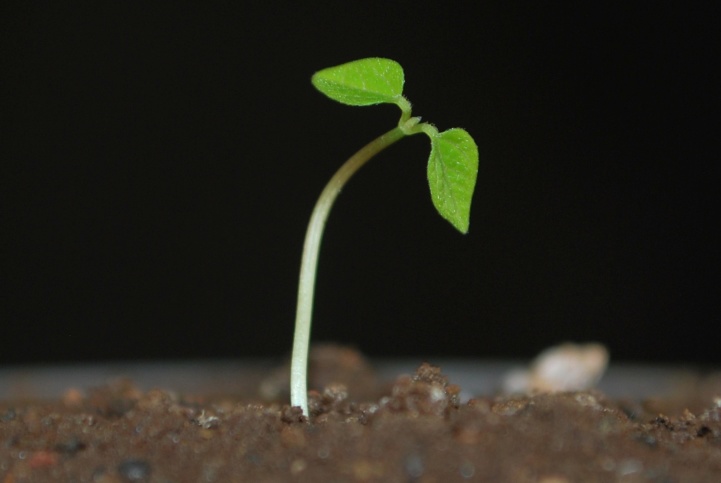
Seedling

**Figure 3f.**
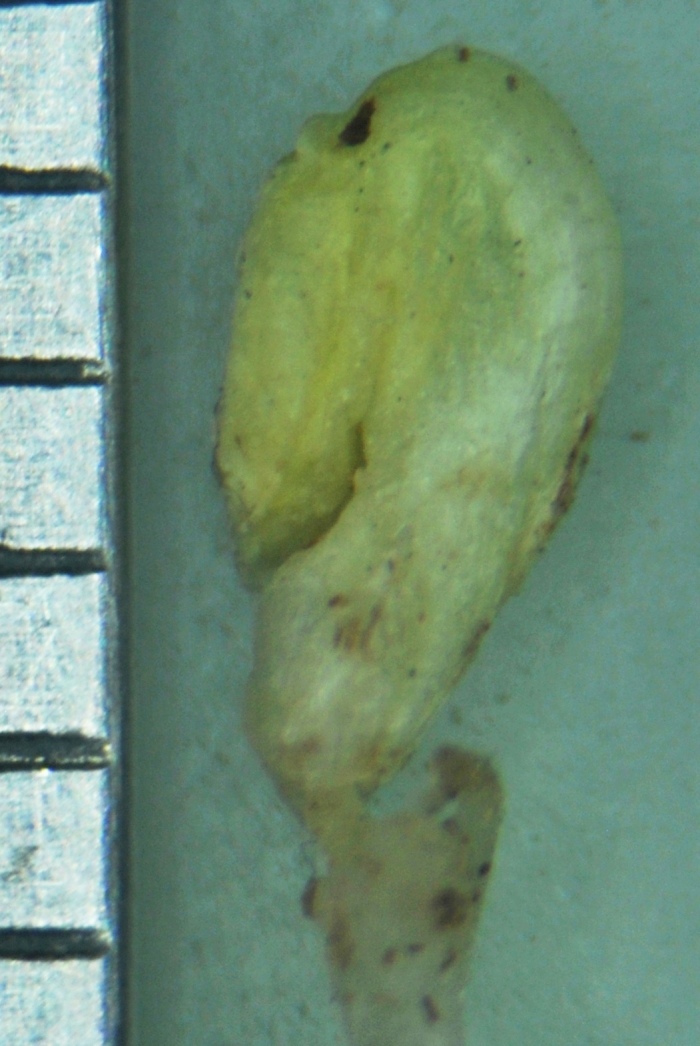
Cleistogamous flower

**Figure 4a.**
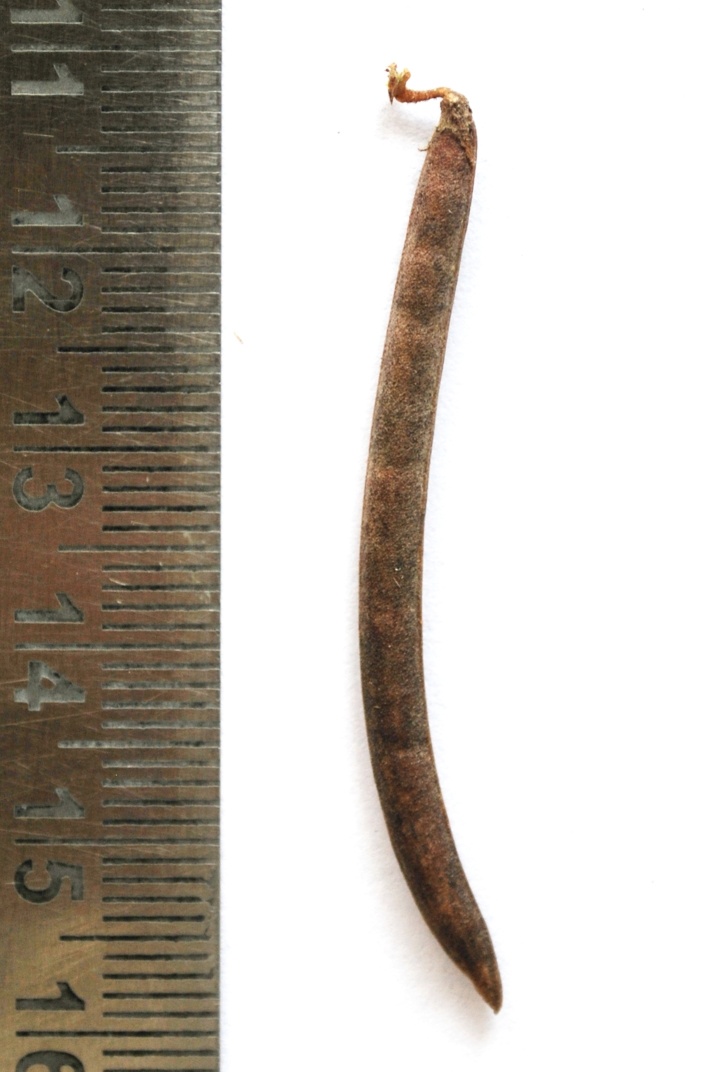
Pod of chasmogamous flower

**Figure 4b.**
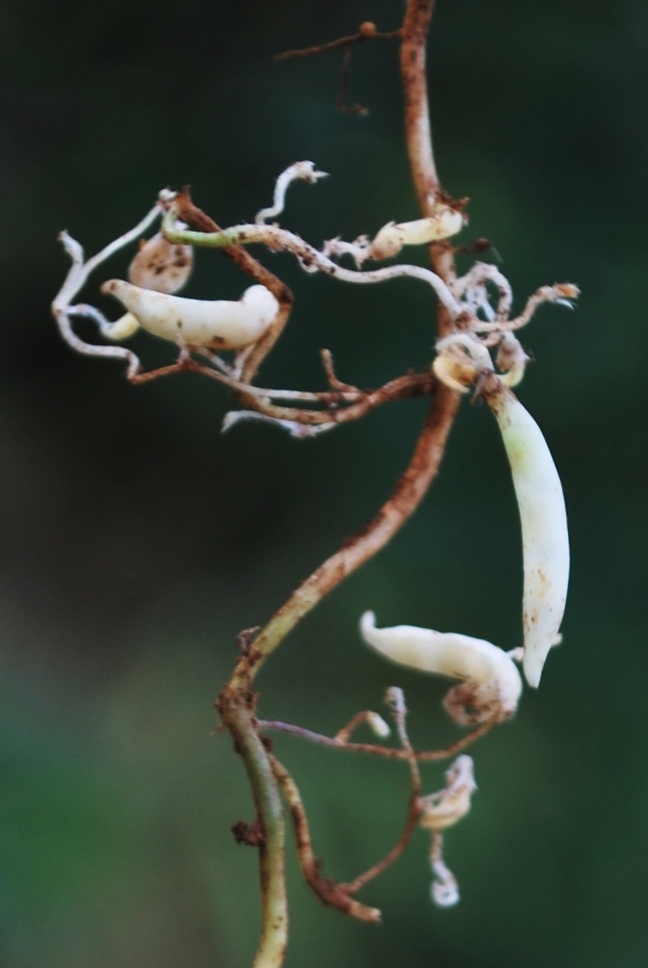
Pods of cleistogamous flowers

**Figure 4c.**
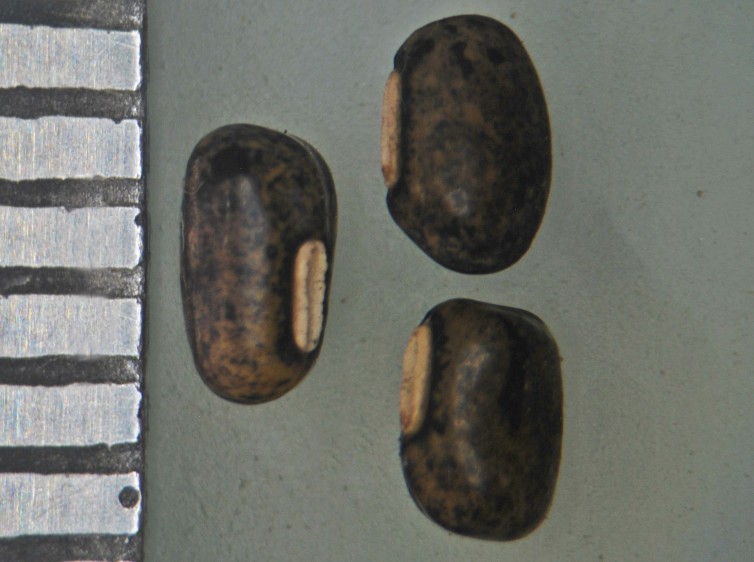
Seeds of the chasmogamous flowers

**Figure 4d.**
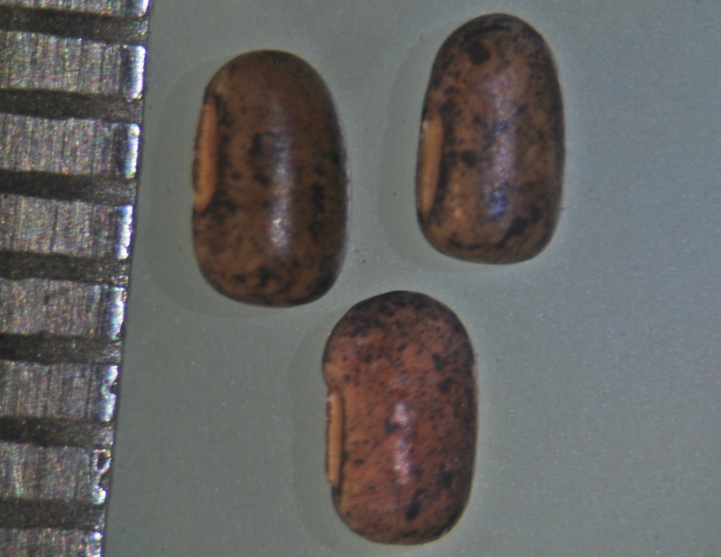
Seeds of the cleistogamous flowers

**Table 1.** Morphological differences between *Vigna yadavii* and *V. dalzelliana*.

**Attributes**	***Vigna yadavii***	***Vigna dalzelliana***
**Chasmogamous flowers**		
Corolla	4–6 mm long	c. 1.5 cm long
Style beak	linear, 0.4–0.5 mm long	flattened, 0.9–1.2 mm long
Calyx	hairy	glabrous
Seeds	6–12 per pod	8–10 per pod
**Cleistogamous flowers**		
Flowers	present on positively geotropic branches	absent
Pods	short, 1.5–2.5 cm long, cylindrical curved, white (albino), glabrescent.	absent
Seeds	3–5 per pod, whitish brown, 2–3 × 1.7–2 mm; hilum poorly developed and not protruded out.	absent

## References

[B925179] Aitawade M. M., Sutar S. P., Rao S. R., Malik S. K., Yadav S. R., Bhat K. V. (2012). Section *Ceratotropis* of subgenus *Ceratotropis* of *Vigna* (Leguminosae–Papilionoideae) in India with a new species from Northern Western Ghats. Rheedea.

[B925194] Babu C. R., Sharma S. K., Johri B. M. (1987). Leguminosae–Papilionoideae: Tribe–Phaseoleae. Bulletin Botanical Survey of India.

[B925204] Delgado-Salinas A., Thulin M., Pasquet R., Weeden N., Lavin M. (2011). *Vigna* (Leguminosae) sensu lato: The names and identities of the American segregate genera. American Journal of Botany.

[B925267] Lewis G. P., Shrine B., Mackinder B., Lock J. M. (2005). Legumes of the World. Royal Botanical Garden Kew, London.

[B925309] Maréchal R., Mascherpa J. M., Stainier F. (1978). Etude taxonomique d’un groupe complex d’espécies des genres Phaseolus et *Vigna* (Papilionaceae) sur la base de données morphologiques et polliniques, traitées par I’anyse informatique. Boissiera.

[B925329] Maxted N., Mabuza-Dalamini P., Moss H., Padulosis S., Jarvis A., Gaurino L. (2004). African *Vigna*: Systematic and Ecogeographic studies. International Plant Genetic Resource Institute Rome, Italy.

[B925431] Tateishi Y. (1984). Contribution to the genus Vigna (Leguminosae). Science Report Tohoku University. Series.

[B925341] Thulin M., Lavin M., Pasquet R., Delgado-Salinas A. (2004). Phylogeny and Biogeography of Wajira (Leguminosae): A Monophyletic Segregate of *Vigna* Centered in the Horn of Africa Region. issn: 0363-6445.

[B1184144] Tomooka Norihiko, Kaga Akito, Isemura Takehisa, Vaughan Duncan, Kole Chittaranjan (2010). *
Vigna
*. Wild Crop Relatives: Genomic and Breeding Resources.

[B925371] Tomooka N., Maxted N., Thavarasook C., Jayasuriya A. H.M. (2002). Two new species, sectional designations and new combinations in *Vigna* subgenus *Ceratotropis* (Piper) Vedc., (Leguminosae, Phaseoleae). Kew Bulletin.

[B1184115] Tomooka Norihiko, Vaughan Duncan A., Moss Helen, Maxted Nigel (2002). The Asian *Vigna*: Genus *Vigna* subgenus *Ceratotropis* genetic resources.

